# Obesity-Related Metabolomic Analysis of Human Subjects in Black Soybean Peptide Intervention Study by Ultraperformance Liquid Chromatography and Quadrupole-Time-of-Flight Mass Spectrometry

**DOI:** 10.1155/2013/874981

**Published:** 2013-06-04

**Authors:** Min Jung Kim, Hye Jeong Yang, Jin Hee Kim, Chang-Won Ahn, Jong Ho Lee, Kang Sung Kim, Dae Young Kwon

**Affiliations:** ^1^Department of Food Metabolism and Nutrition, Korea Food Research Institute, 516 Paekhyon-dong, Pundang-ku, Songnam, Kyongki-do 463-747, Republic of Korea; ^2^Research and Development Center, Nong Shim Co., Ltd., Seoul 156-709, Republic of Korea; ^3^Department of Food & Nutrition, Yonsei University, Seoul 120-749, Republic of Korea; ^4^Department of Food Science & Nutrition, Yongin University, Kyongki-do, 134 Samka-dong, Chuin-ku, Yongin, Kyongki-do 449-714, Republic of Korea

## Abstract

The present study aimed to identify key metabolites related to weight reduction in humans by studying the metabolic profiles of sera obtained from 34 participants who underwent dietary intervention with black soybean peptides (BSP) for 12 weeks. This research is a sequel to our previous work in which the effects of BSP on BMI and blood composition of lipid were investigated. Sera of the study were subjected to ultra performance liquid chromatography and quadrupole time-of-flight mass spectrometry (UPLC-Q-TOF-MS), and the data were analyzed using partial least-squares discriminate analysis (PLS-DA) score plots. Body mass index and percent body fat of the test group were reduced. Levels of betaine, benzoic acid, pyroglutamic acid, pipecolic acid, *N*-phenylacetamide, uric acid, l-aspartyl-l-phenylalanine, and lysophosphatidyl cholines (lysoPCs) (C18:1, C18:2, C20:1, and C20:4) showed significant increases. Levels of l-proline, valine, l-leucine/isoleucine, hypoxanthine, glutamine, l-methionine, phenylpyruvic acid, several carnitine derivatives, and lysoPCs (C14:0, PC16:0, C15:0, C16:0, C17:1, C18:0, and C22:0) were significantly decreased. In particular, lysoPC 16:0 with a VIP value of 12.02 is esteemed to be the most important metabolite for evaluating the differences between the 2 serum samples. Our result confirmed weight-lowering effects of BSP, accompanied by favorable changes in metabolites in the subjects' blood. Therefore, this research enables us to better understand obesity and increases the predictability of the obesity-related risk by studying metabolites present in the blood.

## 1. Introduction

Obesity is considered a perilous disease because, besides being widespread, it is the primary risk factor for the development of various physical, mental, and social disorders such as cardiovascular diseases, type 2 diabetes, obstructive sleep apnea, certain types of cancers, mental depression, and social stigmatization [1, 2]. Causes of obesity are numerous, including imbalance between calorie intake and expenditure, genetic susceptibility, emotional reasons, and environmental factors such as life style [3]. 

Thus appropriate diet and good eating habits are the foundation for physical fitness. Certain Korean traditional fermented soybean foods; fermented soybean and fermented soybean pastes, notably Chungkukjang and Doenjang, may help to alleviate or fight obesity [4–6]. Research results by Yang et al. indicated that feeding chungkukjang decreased blood pressure and serum levels of lipids in rats [7]. Soh et al. tried to identify the weight-reducing effects of fermented soybean products, chungkukjang and doenjang, by analyzing the hepatic mRNA expressions of enzymes related to fatty oxidation [4, 5]. However, most of these research results are unable to correlate substances responsible for antiobesity as soybean and soybean products contain numerous compounds with functional properties, for example, saponins, isoflavones, and proteins [7–10]. 

In addition, to take full advantage of the health-promoting effects of fermented soybean products, physiological effects of purified soybean proteins and soybean peptides are extensively studied by numerous researchers. Nagasawa et al. reported that soybean protein could lower the triglyceride content and fatty acid synthase mRNA level in adipose tissue [11, 12]. In human studies with black soybean peptides (BSPs), Kwak et al. asserted that the peptides were effective in reducing body weight, body fat mass, and plasma leptin levels of obese subjects [13]. Kim et al. were successful in purifying and identifying adipogenesis inhibitory peptides from black soybean protein hydrolysates [14]. 

In our previous paper, we reported that obese research subjects who had taken BSP supplementations experienced significant reduction in body weight, body mass index (BMI), and body fat mass. Serum leptin levels were found to be significantly reduced in these research participants [15]. BSPs used for the experiment are analogues of doenjang (fermented soybean paste) peptides. They were produced by hydrolyzing whole soybean protein with proteases extracted from *Aspergillus oryzae*, the main fungi responsible for doenjang fermentation [16]. The peptide sequences were analyzed to be identical to those of the glycinin fraction of black soybean or to those (data not shown) found in doenjang. Our research group was able to discover that the commonly required structural features for the biological activity of some antiobesity peptides were the hydrophobic moiety and proline [17]. Moreover, identifying specific peptides associated with antiobesity is often difficult because of contaminating peptides with similar physicochemical properties. In another study with humans, we were able to identify 3 lysophosphatidylcholines (lysoPCs), lysoPC C14:0, lysoPC C16:0, and lysoPC C18:0, as the potential markers for overweight/obesity through metabolomic profiling of plasmas of overweight/obese men [18]. Liver and serum metabolites of obese and lean mice fed on high-fat or normal diets revealed that, in addition to lysoPCs, levels of lipid metabolism intermediates such as betaine, carnitine, and acylcarnitines, as well as some branched-chain amino acids (BCAAs), contributed to the discrimination between the 2 groups [19]. 

The aim of the present study is to elucidate the anti-obesity effects of BSPs by studying the metabolic profiles of serum obtained from subjects who have undergone dietary intervention with these peptides. To our knowledge, this is almost the first metabolomic study that attempts to elucidate the weight-reducing effects of soybean peptides in humans. Since metabolites present in the blood directly reflect the body's physiological changes, we expect to obtain further insight into the health-promoting effects of soybean peptides, as well as that of fermented soybean products that have long been known as healthy food in Korea. 

## 2. Materials and Methods 

### 2.1. Subjects Selection and Composition of BSPs

Details on the general characteristics of all volunteers subjects, which were recruited by public advertisement and the diet intervention protocol for obesity by BSP, are provided in our previous paper [15]. The study subjects were 34 overweight/obese subjects aged 19–65, who were interested in losing weights. Both the BSP supplement and placebo were obtained from Nongshim Co., Ltd. (Seoul, Korea). Amino acid analysis showed that BSP, manufactured by hydrolyzing whole soybean proteins with proteases from *Aspergillus oryzae* was composed of low-molecular weight amino acids of less than 10,000 kDa. Some of the peptide sequences analyzed by LC-MS were as follows: NLQGENEEEDSGAIVTVK, VSIIDTNSLENQLDQMPR, KEQQQEQQQEEQPLEVR, EQQQEQQQEEQPLEVR, GNPDIEHPETM, LDTSNFNNQLDQTPRVF, NQEQEFLKYQ, RLLLLLGWLLIIVGVILLVGSTK, KEQQQEEQQEEQPLEVR, IIDTNSLENQLDQMPR, LDTSNFNNQLDQNPRVF, EQQQRQQQEEQPLE, and PMDYYSDYDDNADDYFDDADDSDR (see supplement data are available online at http://dx.doi.org/10.1155/2013/874981). These amino acid sequences coincided with those of glycinin subunits present in black soybean (http://www.ncbi.nlm.nih.gov/). 

### 2.2. Sample Preparation for Analysis

We collected 150 *μ*L of serum from the 34 study participants to which 300 *μ*L of cold acetonitrile was added to extract soluble metabolites. After shaking for 30 min at 4°C, the samples were centrifuged at 10,000 rpm for 10 min at 4°C. The supernatants, which were freeze-dried and stored at −70°C, were dissolved in 20% methanol just before ultra performance liquid chromatography and quadrupole time-of-flight mass spectrometry (UPLC-Q-TOF-MS) analysis [17, 20]. 

### 2.3. Characteristics of Participants

The characteristics of the participants observed in previous papers [15] are described in short as follows: all subjects who participated in the present research project were healthy individuals without any signs of apparent ailments such as heart diseases or diabetes (Table 1). Moreover, according to the criteria of the Korean Society of the Study for Obesity (KSSO), most of the participants were classified as overweight and obese (BMI value of 23–25 and ≤25, resp.). Mean age, BMI (*P* < 0.001), and percent body fat (*P* = 0.002) of the test group consisting of 34 subjects (15 men and 19 women) were 39.0 ± 1.74, 28.0 ± 0.47, and 32.0 ± 1.06, respectively. Total energy intake (*P* < 0.001) and expenditure (*P* < 0.001) of the test group were 2, 518 ± 61.5 kcal/day and 2, 567 ± 63.6 kcal/day during the 12 weeks of testing, respectively. Carbohydrates comprised the largest portion of energy intake (61.5–62.0%), followed by fat and protein. No noticeable differences in eating behavior were apparent between before and during the research period, except for the individuals in the test group, who were taking one packet of dietary supplements containing 4.5 g of BSP after each meal.

### 2.4. Serum Analysis by UPLC-Q-TOF-MS

Serum extracts were analyzed on a UPLC-Q-TOF-MS instrument (Waters, Milford, MA, USA), as described by Kim et al. [17]. The Q-TOF-MS was operated in positive electrospray ionization (ESI) mode, with a scan range of *m/z* 50–1,000. Cone voltage was 30 V, capillary voltage was 3 kV, and scan time was 0.2 s, with an interscan delay of 0.02 s. The source temperature was set at 110°C, while the desolvation flow was set at 700 L/h; the desolvation gas temperature was set at 300°C. The MS was calibrated using sodium formate, and leucine enkephalin was used as lock mass. The concentration of leucine enkephalin was 200 *ρ*M and the flow rate was set at 5 *μ*L/min. As quality control, a mixture of 5 standard compounds (4-acetoaminophenol, caffeine, sulfadimethoxine, terfenadine, and reserpine) was injected after running every 8 samples. 

In the MS-MS experiments, argon was used as collision gas, with the collision energy alternating between 10 and 30 eV. The MassLynx software version 4.1 (Waters Inc.) was used to control the instrument and calculate accurate masses. Peaks were collected using a peak width at 5% height, 1 s, a noise elimination of 6, and an intensity threshold of 70. Data were aligned with a mass tolerance of 0.04 Da and a retention time window of 0.2 min. All spectra were aligned and normalized to an external standard. Assignment of metabolites contributing to the observed variance was performed using the ChemSpider (http://www.chemspider.com/) and Human Metabolome Database (http://www.hmdb.ca/).

### 2.5. Data Processing for Statistical Analyses

Statistical analyses were performed on the data using SIMCA-P+ software (version 12.0.1, Umetrics Inc., Umeå, Sweden). Partial least-squares discriminant analysis (PLS-DA) was used to visualize discrimination among samples and an internal 7-fold cross-validation was carried out to estimate the performance of the PLS-DA models. Goodness of the fit was quantified by *R* ^2^ *X* and *R* ^2^ *Y* and the predictive ability was indicated by *Q* ^2^ *Y*. In addition to cross-validation, model validation was also performed by a 200 times permutation test. Analysis of variance was performed to determine statistical significance by using SPSS 11.5 (SPSS Inc., Chicago, IL, USA) at a significance level of *P* < 0.05 [17, 19].

## 3. Results and Discussion

### 3.1. Characteristics of Participants after Dietary Intervention

After completion of the test period, BMI and percent body fat of the test group were found to be 27.6 ± 0.48 and 31.3 ± 1.07%, respectively. Both indices were consistently lower compared to the start of the intervention, suggesting effectiveness of soybean peptides on weight reduction. Triglyceride levels of the test group were lower after 12 weeks. Initial and final serum triglyceride concentrations of the test group were 134.7 ± 13.7 mg/dL and 123.0 ± 12.8 mg/dL, respectively, (*P* < 0.1). Total cholesterol levels (*P* < 0.001) were almost unchanged at start and the end of the trial. In contrast to the total cholesterol concentration, HDL-cholesterol levels (*P* < 0.001) exhibited a marked increase at the end of the dietary intervention. The concentration of the macromolecular complex increased from 33.7 ± 1.37 mg/dL to 39.3 ± 1.77 mg/dL. 

Our result in good agreement with other similar nutritional intervention studies in which the effect of soybean protein on mice or humans was investigated [21–23]. Rho et al. revealed that mice with diet-induced obesity-induced mice showed reduced body weight as well as improved lipid profiles when fed with BSP for 28 days, as compared to casein-fed mice [21]. Jang et al. also proved that BSP has anti-obesity and triglyceride-reducing effects on mice fed with a high-fat diet for 13 weeks [22]. In human studies with soybean-based meals, Allison et al. showed that 100 obese people exhibited significantly reduced body weight, body fat mass, total cholesterol, and LDL cholesterol after participation in a randomized controlled trial for 12 weeks [23]. The results obtained here indicate that our intervention study could be a good example for further human metabolomics studies.

### 3.2. Serum Metabolic Profile

**Figure 1:**
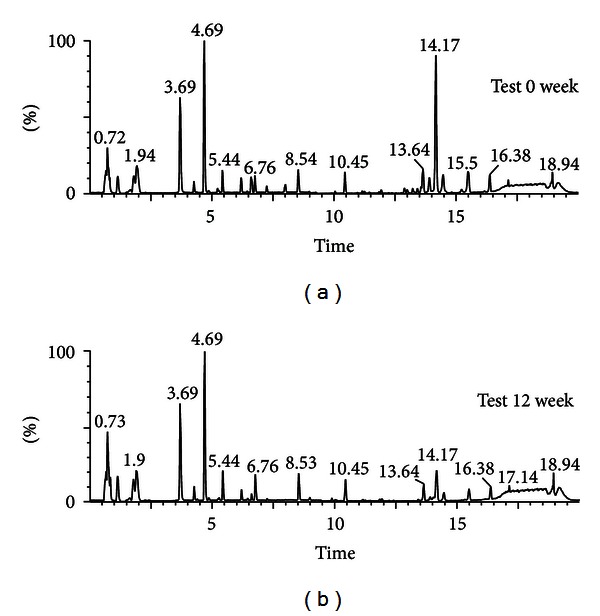
Ultraperformance liquid chromatography-quadrupole-time-of-flight mass spectrometry (UPLC-Q-TOF MS) profiles of sera from human subjects who were controlled by BSPs before and after 12 weeks of intervention.

**Figure 2:**
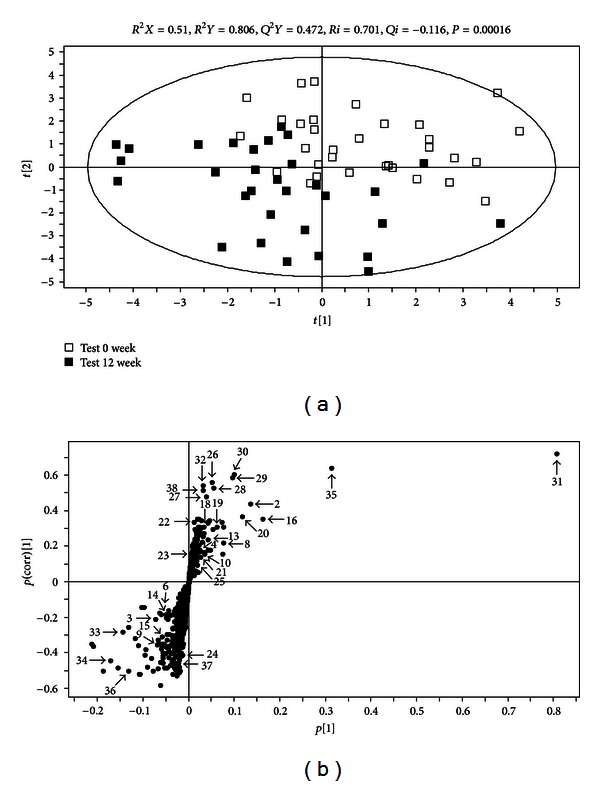
(a) Partial least-squares discriminant analysis (PLS-DA) scores plot (a) obtained from the mass spectrometry data of the sera from study subjects. Data allowed clear discrimination between the subjects at week 0 (open square) and at week 12 (filled square). Outlying samples of the ellipse region with the 95% confidence interval were excluded by the Hotelling's *T* ^2^ test. *R* ^2^ *X*, *R* ^2^ *Y*, and *Q* ^2^ *Y*. The PLS-DA model was validated by a permutation test: *P*-values and intercepts of *R* ^2^ (*Ri*) and *Q* ^2^ (*Qi*). (b) S-plot covariance [*p*] and reliability correlation [*p*(corr)] from PLS-DA models (b) and loadings plots (b). The numbers for the metabolites are as given in Table 2.

Sera of the participants in the test group were collected at the initial and final stages of the diet intervention and analyzed by UPLC-MS. The resulting base peak intensity chromatograms obtained in positive ion (ESI^+^) mode are shown in Figure 1. Next the obtained data were applied to a PLS-DA score plot to elucidate the existence of class distinction between the stages. The PLS-DA score plot showed a separation between 0 week and 12 week samples along the axes corresponding to the first 2 PLS-DA components (Figure 2(a)). The variation in *X* (*R*_2_ *X*) was 51.0%, while the variation in *Y* (*R*_2_ *Y*) was 80.6% which predicts 47.2% of the variation in response to *Y* (*Q* ^2^ *Y* = 47.2%) for 2-component model. The permutation test with a permutation number of 200 was performed and indicated a *R* ^2^ intercept value of 0.701 and a *Q* ^2^ intercept value of −0.116. Thus, the result shows that 0-week samples and 12-week samples could be clearly differentiated from each other by the primary or secondary component with the goodness of fit of the data. This result indicated that the study subjects who took BSP for the period of 12 weeks experienced changes in the profiles of the serum metabolites. 

### 3.3. Identification of Possible Biomarkers

The S plot of metabolites along the axes corresponding to the combined weight and reliability correlation (*p*(corr)) indicates the contribution of individual metabolites to the separation of the 2 samples. The higher or lower the value of *p*(corr) of the metabolites, the greater the degree of contribution made by these metabolites to the discrimination of the test samples. Metabolites with positive *p*(corr) values correspond to those with decreased serum levels caused by diet intervention, whereas metabolites with negative value correspond to those whose level increased during the study period of 12 weeks. All 758 metabolites detected with our UPLC-MS system are shown in the S plot (Figure 2(b)), and the metabolites with marked numbers are the ones that were identified using MS-MS. Variable importance in the projection scores (VIP scores) as well as the normalized fold changes of the metabolites due to dietary intervention is also presented in Table 2. The normalized levels of the metabolites in the sera collected at weeks 0 and 12 of the intervention were statistically analyzed by using the *t*-test. 

**Figure 3:**
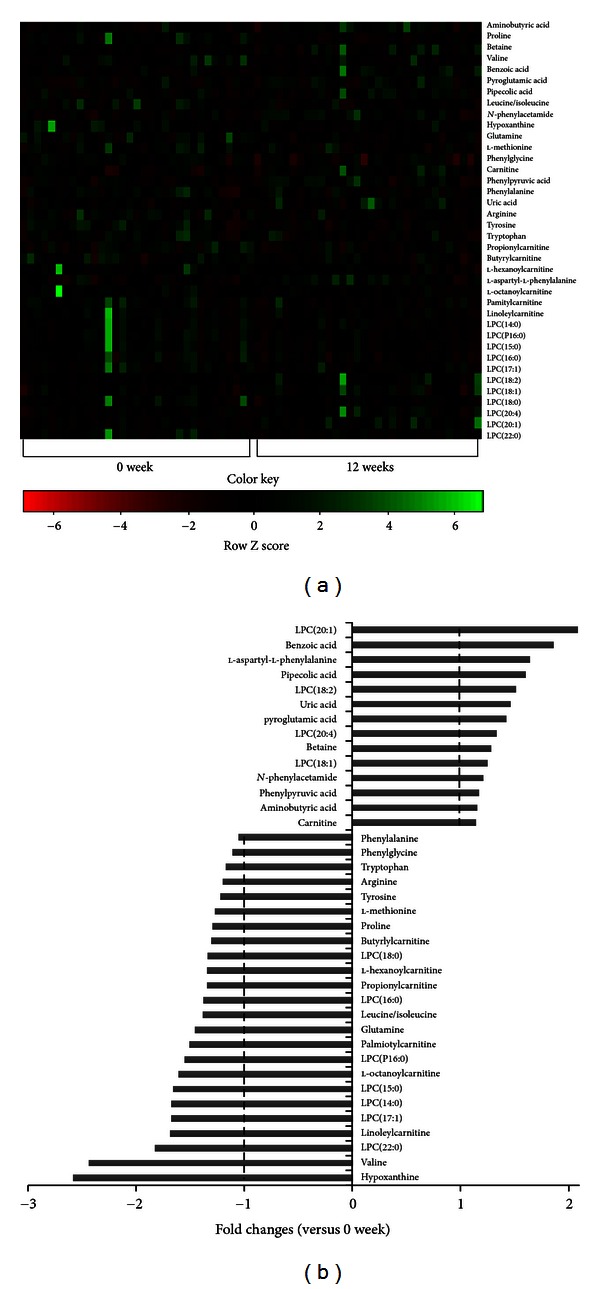
Heat map of the identified serum metabolites from the human subjects showing significant differences among samples (a) and their fold changes at weeks 0 and 12 of intervention (b). The heat map was drawn by *R* with g plots. The fold changes of serum metabolites in subjects at 12 weeks were calculated against those at 0 week and are presented as positive and negative values.

**Figure 4:**
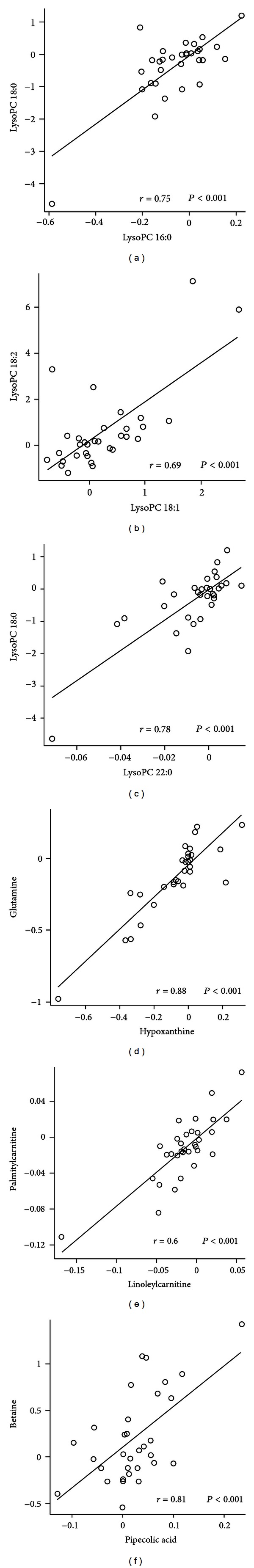
Relationship between the changes in the major metabolite levels by Pearson's correlation analysis. *r*, correlation coefficient. The changes in lysophosphatidylcholine (lysoPC) 16:0 levels were positively related to those of lysoPC 15:0 (*r* = 0.90, *P* < 0.001) and lysoPC 18:0 (*r* = 0.76, *P* < 0.001), while that of lysoPC 18:0 was closely related to that of lysoPC 15:0 (*r* = 0.77, *P* < 0.001). The change in the lysoPC 18:2 level was closely related to that of lysoPC 18:0 (*r* = 0.81, *P* < 0.001), while that of lysoPC 18:1 was related to that of lysoPC 18:2 (*r* = 0.69, *P* < 0.001). The change in the proline level was related to those of tryptophan (*r* = 0.53, *P* < 0.001) and phenylalanine (*r* = 0.55, *P* < 0.001).

Eleven serum metabolites, that is, betaine (*P* = 0.026), benzoic acid (*P* = 0.024), pyroglutamic acid (*P* = 0.021), pipecolic acid (*P* = 0.042), *N*-phenylacetamide (*P* = 0.011), uric acid (*P* = 0.02), l-aspartyl-l-phenylalanine (*P* = 0.0002), and lysoPCs containing C18:1 (*P* = 0.037), C18:2 (*P* = 0.045), C20:1 (*P* = 0.008), and C20:4 (*P* = 0.03), showed a significant increase during 12 weeks of peptide intervention, whereas 20 metabolites, that is, l-proline (*P* = 0.003), valine (*P* = 0.042), l-leucine/isoleucine (*P* = 0.048), hypoxanthine (*P* = 0.036), glutamine (*P* = 0.028), l-methionine (*P* = 0.039), phenylpyruvic acid (*P* = 0.041), propionylcarnitine (*P* = 0.05), butyrylcarnitine (*P* = 0.05), l-hexanoylcarnitine (*P* = 0.06), l-octanoylcarnitine (*P* = 0.014), palmiotylcarnitine (*P* = 0.034), linoleylcarnitine (*P* = 0.02), and PCs containing C14:0 (*P* = 0.045), PC16:0 (*P* = 0.02), C15:0 (*P* = 0.012), C16:0 (*P* = 0.018), C17:1 (*P* = 0.02), C18:0 (*P* = 0.038), and C22:0 (*P* = 0.029) showed decreased levels. Aminobutyric acid (*P* = 0.04), carnitine (*P* = 0.02), *N*-phenylacetamide (*P* = 0.011), 2-phenylglycine (*P* = 0.013), phenylalanine (*P* = 0.045), arginine (*P* = 0.044), tyrosine (*P* = 0.01), and tryptophan (*P* = 0.043) showed little changes in the concentration with fold changes being in the range of 0.8–1.2. Among 38 metabolites identified, l-proline, betaine, and lysoPCs containing C16:0, C18:0, C18:1, and C20:1 exhibited VIP values greater than 3.0, indicating a high correlation with discrimination between the samples collected at weeks 0 and 12 of diet intervention. In particular, lysoPC 16:0 with a VIP value of 12.02 is esteemed to be the most important metabolite for evaluating the differences between the 2 serum samples (Figure 3). A similar result was revealed by Kim et al. who showed that, in mice with diet-induced obesity, lysoPC 16:0 was the single most important metabolite related to obesity [17]. Using Pearson's correlation analysis, relationships between the changes in the levels of major metabolites were elucidated and are shown in Figure 4. The changes in lysoPC 16:0 levels were positively related to lysoPC 18:0 (*r* = 0.751, *P* < 0.001), while that of lysoPC 18:1 was found to be related to lysoPC 18:2 (*r* = 0.690, *P* < 0.001). The change in the lysoPC 22:0 level was closely related to lysoPC 18:0 (*r* = 0.789, *P* < 0.001). The change in the hypoxanthine level was related to glutamine (*r* = 886, *P* < 0.001). The change in levels of linoleylcarnitine and pipecolic acid was related to palmityl carnitine (*r* = 0.602, *P* < 0.001) and betaine (*r* = 0.810, *P* < 0.001), respectively. Obesity is known to be accompanied by distinct changes in the metabolite composition of serum, including lipids, amino acids and other small compounds. Of particular interest is lysoPC, as numerous studies have suggested that lysoPC levels are closely associated with ailments such as endothelial dysfunction, inflammation, and atherogenesis, as well as obesity [24–26]. Since lysoPC has a relatively short half-life, it is thought to be metabolic intermediates that are produced during the formation or breakdown of other lipids. These compounds are formed in the liver and carried by albumin and lipoproteins to various parts of the body. According to Croset et al., unsaturated lysoPCs are associated with albumin rather than lipoproteins [27]. LysoPCs are known to account for 5–20% of all phospholipids in the serum [28]. Our studies showed that sera of subjects after 12 weeks of diet intervention exhibited slight reductions in the levels of both total triglycerides and percent body fat though those of lysoPCs did not show a definite trend and were shown to be dependent on the types of fatty acid chains esterified to the glycerol moiety. A positive relationship existed between the changes in the levels of lysoPCs with saturated fatty acids at *sn*-2 position of phosphatidylcholine, while the levels of lysoPCs with unsaturated fatty acid esterified at *sn*-2 position increased during the diet intervention. Our result is in accordance with those of other studies with of obese men, pigs, and monozygotic twins [19, 24, 29]. The serum levels of lysoPCs 14:0 and 18:0 in obese men and lysoPC18:0 in pigs with diet-induced obesity were higher than those of controls, whereas that of lysoPC 18:1 was decreased in obese men, while no change was observed in the lysoPC 16:0 level in pigs [24]. The studies with monozygotic twins showed a close correlation between increased lysoPC levels and obesity [29]. However, other studies showed that the relationship between lysoPC levels and obesity is unclear and that further studies are needed to elucidate the role of lysoPCs in weight reduction [24]. 

Betaine is another compound that was found to be increased in the serum of participants during the diet intervention program. In addition to its function as an organic osmolyte and methyl donor in the remethylation step of the methionine-homocysteine cycle, betaine is known to prevent or reduce the accumulation of fat, especially in the liver, and, thus, is called a lipotrope [30]. Researchers have found that betaine helps to reduce hepatic cholesterol and phospholipids in rats fed a high-fat diet [31], relieve hyperlipidemia [32], and increase the production of carnitine [33]. These findings might explain why betaine levels were increased in subjects who had undergone dietary intervention with BSP. Nevertheless, mechanisms behind elevation of betaine level following BSP consumption are uncertain at this moment and thus need further researches. 

The levels of carnitine were found to be increased by dietary intervention, too. However those of acylcarnitines, that is, propionylcarnitine, butyrylcarnitine, l-hexanoylcarnitine, l-octanoylcarnitine, palmitylcarnitine, and linoleylcarnitine, were decreased. Carnitine is a quaternary ammonium compound that is naturally produced in the human body from l-lysine and l-methionine or supplied through dietary sources [34]. Carnitine synthesis is known to occur both in the kidney and liver and carnitine is transported in the blood for use by muscles. Acylcarnitines are synthesized in the cytosol, which then enter the mitochondria where the acyl group is removed from carnitine to form acyl-CoA. The acyl group is subsequently catabolized in 2-carbon units by *β*-oxidation inside the mitochondrial matrix. Thus, carnitine and acylcarnitines are considered important biomarkers for obesity ([35], see http://en.wikipedia.org/wiki/Carnitine). Reports indicate that carnitine can diminish the risk of obesity caused by a high-fat diet in mice [36] or in obese humans [37]. Some studies show that carnitine might indirectly work to reduce the weight in obese subjects by increases in muscle mass or reduction in fatigue [38].

One of the possible reasons for the increased levels of carnitine in our study might be due to the relatively high amounts of precursor amino acids present in the dietary supplements taken by the participants for 12 weeks. Moreover, the decreased levels of acylcarnitines were quite unexpected and, thus, further studies are needed to better understand the study outcome. One of the possible reasons for the observed effect might be the reduction in percent body fat and serum triglyceride levels in our test subjects, which could have resulted in a decrease in the levels of acylcarnitines, as these compounds are substrates for the biosynthesis of acylcarnitines. Rho et al. assert that the depletion of fatty acids due to energy production via *β*-oxidation and increased citric acid cycle activity might lead to decreased transportation of fatty acids in adipose tissue in participants who had taken BSP along with their normal diet [21]. 

The levels of BCAAs notably valine and leucine/isoleucine were found to be lower after diet intervention, along with those of aromatic amino acids such as tyrosine, phenylalanine, tryptophan, and other amino acids, for example, arginine, l-methionine, proline, and l-glutamine. The decrease in the levels of BCCAs and aromatic amino acids with weight loss is consistent with recent findings where close relationships were established between changes in blood BCAAs levels and obesity (http://www.umm.edu/altmed/articles/carnitine-l-000291.htm). However, the underlying mechanisms of the relationship between obesity and increased BCAA levels is not yet fully understood. BCAA levels might increase simply because obese individuals eat more or because of increased protein catabolism as the result of insulin resistance. Jensen and Haymond [39] reported that proteolysis is increased in obese individuals, whom the antiproteolytic action of insulin is impaired. Two studies, one with obese and lean humans, and the other with animals on high-fat diet with and without BCAA supplementation or on normal diet revealed that blood BCAA levels were consistently higher in obese subjects. The level of valine is especially associated with a decreased BCAA catabolism rate which is attributed to the development of obesity-associated insulin resistance. According to a study by Kim et al., higher levels of tyrosine and tryptophan (or, with regard to our study, the decreased levels of aromatic amino acids in subjects who had undergone diet intervention) in obese individuals might be due to their competition for transport into cells with large neutral amino acids [19]. 

Uric acid is synthesized by the enzyme xanthine oxidase; xanthine and hypoxanthine serve as substrates. Among several metabolites, uric acid levels were increased in the blood of study participants, while those of hypoxanthine were significantly decreased. Since all of our study participants experienced a decrease in body weight, percent body fat, and blood triglyceride levels, this result is unexpected as uric acid is widely known to be an indicator for obesity. Several reports asserted that elevated uric acid levels (as well as those of hypoxanthine) are closely related to an increase in body weight and body fat as well as obesity-related diseases such as diabetes, abdominal obesity, endothelial dysfunction, inflammation, and subclinical atherosclerosis [40]. Thus, further studies are needed to clarify the differences between our results and those of others. In addition, the roles of other metabolites identified in this research, for example, benzoic acid, l-aspartyl-l-phenylalanine, pipecolic acid, pyroglutamic acid, *N*-phenylacetamide, phenylpyruvate, and aminobutyric acids, which were all increased as the result of BSP consumption are not understood at this time and, thus, requires further studies to elucidate the underlying mechanism. We already suggested some mechanisms of ageing and obesity by proposing regulating pathways in mice with metabolomics approaches [17, 41]. In this study, however, the data are not sufficient to discuss the mechanisms of the BSP-induced antiobesity effect in humans. Further studies are needed to accumulate data for studying the antiobesity mechanisms for some nutraceuticals.

Numerous beneficial functions of soybean peptides have been identified, including hypolipidemic effects, improvement in endothelial function, insulin resistance, and weight loss. Since obesity is associated with the development of some major human diseases, it is considered a serious problem worldwide. The main cause of obesity is the imbalance between energy intake and energy expenditure, which leads to an increase in body weight and percent body fat. Nagasawa et al. showed that soy protein isolates control the gene expression in adipose tissue and effectively regulate adipogenesis, which results in a lower triglyceride content in adipose tissue [11, 12]. Our result confirmed weight-lowering effects of BSP, accompanied by favorable changes in metabolite levels in the blood of subjects who have participated in this research. Therefore, the results of this study allow us to better understand obesity and its related diseases, increase the predictability of obesity-related risks by studying metabolites present in the blood, and assess the therapeutic effects of antiobesity agents.

## Supplementary Material

Supplementary Figure: Mass spectra of peptides from black soybean digested by proteases from *Aspergillus oryzae*. The spectra are interpreted in terms of b-type fragment ions using Accurate-Mass Q-TOF LC/MS (Agilent Technologies, DE) with HPLC. Peptide sequence represented by one-letter amino acid abbreviations for each peptide is shown at the top of each spectra.

## Figures and Tables

**Table 1:** Characteristics of BSP (black soybean peptides) controlled subjects before and after 12-week interventions^a^ (data were adopted from Tables  1 and 2 in previous paper (1)).

	0 week	12 week
BMI (kg/m^2^)	28.0 ± 0.47	27.6 ± 0.48
Body fat (%)	32.0 ± 1.06	31.3 ± 1.07
Energy intake and expenditure		
TEE (kcal)	2514 ± 67.0	2567 ± 63.6
TCI (kcal/d)	2560 ± 59.7	2518 ± 61.5
TG (mg/dL)^b^	134.7 ± 13.7	123.0 ± 12.8
T-chol (mg/dL)	171.7 ± 7.40	173.2 ± 7.24
HDL-chol (mg/dL)	33.7 ± 1.37	39.3 ± 1.77

^a^Mean ± SEM. ^b^Tested by long transformed. BMI: body mass index, TEE: total energy expenditure, TCI: total calorie intake, TG: triglyceride, T-chol: total cholesterol, HDL-chol: high-density lipoprotein cholesterol.

**Table 2:** Identification of serum metabolites from human subjects controlled by obesity with BSP using UPLC-MS and their fold change analysis.^a^

No.	Identity	Exact mass	Actual mass	Mass error	Ms fragments	*P* value	VIP
		(M + H)	(M + H)	(mDa)			
1	Aminobutyric acid	104.0712	104.0723	−1.1	104, 87, 58	0.04	0.66
2	l-proline	116.0712	116.0718	−0.6	70	0.03	3.98
3	Betaine	118.0868	118.0878	−1.0	118, 58	0.026	3.23
4	Valine	118.0868	118.0901	−3.3	72	0.042	0.96
5	Benzoic acid	123.0434	123.0462	−2.8	95, 79	0.024	0.49
6	Pyroglutamic acid	130.0504	130.0521	−1.7	84, 72	0.021	1.81
7	Pipecolic acid	130.0868	130.0880	−1.2	105, 91, 84	0.042	1.54
8	Leucine/isoleucine	132.1025	132.1034	−0.9	119, 91, 86, 72, 69	0.048	2.91
9	*N*-phenylacetamide	136.0762	136.0777	−1.5	119, 107, 91	0.011	0.30
10	Hypoxanthine	137.0463	137.0475	−1.2	119, 110, 94, 82	0.036	2.29
11	Glutamine	147.0770	147.0784	−1.4	130, 101, 84	0.028	1.17
12	l-methionine	150.0589	150.0602	−1.3	133, 104, 87, 74, 61	0.039	1.07
13	2-phenylglycine	152.0712	152.0719	−0.7	105, 78	0.013	2.00
14	l-carnitine	162.1130	162.1140	−1.0	103, 85, 60	0.02	1.77
15	Phenylpyruvic acid	165.0552	165.0565	−1.3	147, 123, 119, 91.77	0.041	2.16
16	Phenylalanine	166.0868	166.0883	−1.5	121, 120, 103, 93	0.045	1.58
17	Uric acid	169.0362	169.0991	−62.9	169, 152, 141, 126, 70	0.02	0.49
18	Arginine	175.1195	175.1208	−1.3	130, 116, 70, 60	0.044	0.70
19	Tyrosine	182.0817	182.0830	−1.3	165, 136, 123, 91	0.01	2.58
20	Tryptophan	205.0977	205.0994	−1.7	188, 159, 146, 118, 91	0.043	2.56
21	Propionylcarnitine	218.1392	218.1406	−1.4	159, 144, 85, 60	0.05	1.35
22	Butyrylcarnitine	232.1549	232.1564	−1.5	217, 173, 144, 113, 85	0.05	1.15
23	l-hexanoylcarnitine	260.1858	260.1875	−1.7	232, 201, 144, 85	0.06	0.83
24	l-aspartyl-l-phenylalanine	281.1137	281.1152	−1.5	235, 166, 120, 88	0.0002	1.62
25	l-octanoylcarnitine	288.2170	288.2185	−1.5	229, 127, 85	0.014	2.34
26	Palmitoylcarnitine	400.3427	400.3441	−1.4	341, 144, 85	0.034	0.85
27	Linoleylcarnitine	424.3427	424.3433	−0.6	352, 144, 85	0.02	0.87
28	LysoPC (14:0)	468.3090	468.3092	−0.2	450, 357, 285, 184, 104, 86	0.045	0.99
29	LysoPC (P 16:0)	480.3454	480.3424	3.0	339, 240, 184, 104, 86	0.02	1.80
30	LysoPC (15:0)	482.3247	482.3256	−0.9	385, 299, 184, 104, 86	0.012	1.95
31	LysoPC (16:0)	496.3403	496.3407	−0.4	478, 313, 258, 184, 104, 86	0.018	12.02
32	LysoPC (17:1)	508.3767	508.3429	33.8	492, 327, 258, 184, 104, 86	0.02	0.55
33	LysoPC (18:2)	520.3403	520.3379	2.4	502, 337, 258, 184, 104, 86	0.045	5.48
34	LysoPC (18:1)	522.3560	522.3513	4.7	504, 339, 258, 184, 104, 86	0.037	3.63
35	LysoPC (18:0)	524.3716	524.3672	4.4	506, 341, 258, 184, 104, 86	0.036	5.25
36	LysoPC (20:4)	544.3403	544.3400	0.3	361, 184, 104, 86	0.03	2.48
37	LysoPC (20:1)	550.3873	550.3895	−2.2	532, 418, 258, 184, 104, 86	0.008	0.64
38	LysoPC (22:0)	572.3716	572.3731	−1.5	554, 295, 184, 104, 86	0.029	0.58

^a^No. was the number of metabolites marked in Figure 2(b), and fold change was calculated by dividing the mean of normalized intensities of each metabolite from 12 weeks after subjects by the mean intensity of the same metabolite from 0 week subjects. *P*-value was analyzed by independent *t*-test with the Mann-Whitney *U*-test. VIP is variable importance in the project and its value of above 1.00 showing high relevance for explaining the differences of sample groups.
