# Supplementary material for: Obesity-Related Metabolomic Analysis of Human Subjects in Black Soybean Peptide Intervention Study by Ultraperformance Liquid Chromatography and Quadrupole-Time-of-Flight Mass Spectrometry
Source: J Obes. 2013 Jun 4;2013:874981. doi: 10.1155/2013/874981 (PMC3686146; doi:10.1155/2013/874981)
Supplement: Supplementary file 1 — Supplementary Figure: Mass spectra of peptides from black soybean digested by proteases from Aspergillus oryzae. The spectra are interpreted in terms of b-type fragment ions using Accurate-Mass Q-TOF LC/MS (Agilent Technologies, DE) with HPLC. Peptide sequence represented by one-letter amino acid abbreviations for each peptide is shown at the top of each spectra. [file 874981.f1.docx]

**Supplement data**

Supplementary Figure: Mass spectra of peptides from black soybean digested by proteases from *Aspergillus oryzae*. The spectra are interpreted in terms of b-type fragment ions using Accurate-Mass Q-TOF LC/MS (Agilent Technologies, DE) with HPLC. Peptide sequence represented by one-letter amino acid abbreviations for each peptide is shown at the top of each spectra.
